# Efficacy of Ologen matrix implant in Ahmed Glaucoma Valve Implantation

**DOI:** 10.1038/s41598-019-38656-x

**Published:** 2019-02-28

**Authors:** Marina Sastre-Ibáñez, Carmen Cabarga, María Isabel Canut, Francisco Pérez-Bartolomé, J. L. Urcelay-Segura, R. Cordero-Ros, Julián García-Feijóo, Jose María Martínez-de-la-Casa

**Affiliations:** 1 Ophthalmology Department, Clínico San Carlos Hospital, Ophthalmology Department, Medicine Faculty, Complutense de Madrid University, and Instituto de Investigación Sanitaria del Clínico San Carlos Hospital (IdISSC), Madrid, Spain; 2 Ophthalmology Department, Ramón y Cajal Universitary Hospital, Madrid, Spain; 3 Glaucoma Department, Centro de Oftalmología Barraquer, Barcelona, Spain; 4 Glaucoma Department, Gregorio Marañón Universitary Hospital, Madrid, Spain; 5 Glaucoma Department, La Paz Universitary Hospital, Madrid, Spain

## Abstract

To determine the efficacy and safety of the Ologen collagen matrix adjunctive to Ahmed valve surgery. A randomized prospective multicentre clinical trial involving 58 patients that were followed for one year. Conventional surgery with Ahmed valve was performed in 31 eyes (Control group/CG) and in 27 Ologen (Ologen group/OG) was placed over the valve’s plate. Baseline data: age, corneal thickness, intraocular pressure(IOP) and antiglaucoma medications.Postoperative data (days 1, 7 and months 1, 3, 6 and 12): IOP, antiglaucoma medications, visual acuity and complications were recorded. Frequency of hypertensive phase, complete and qualified success and survival rate were studied. No differences were found between CG and OG in the baseline data. The only difference between groups was a significantly lower IOP at day 1. No other differences were found in the follow-up between groups. Hypertensive phase (56%CG and 55%OG, p = 0,947), complete success 28,6%CG and 30,4%OG (p = 0,88) and qualified success 96,4% and 95,9%(p = 0,794). Survival rates at 1 year were 76,7%(CG) and 69,2%(OG)(p = 0,531). 38,7% of patients in the CG suffered some complication during follow-up and 61,5% in OG(p = 0,086). Ologen does not increase safety or efficacy in Ahmed valve surgery at one-year follow-up. This is the first study that shows no benefit of Ologen adjunctive to this surgery.

## Introduction

Glaucoma drainage devices (GDD) are commonly used in glaucoma surgery specially after the TVT (Tube versus Trabeculectomy) study that showed a higher success rate and lower rate of re-operations of tube shunt surgery (Baerveldt 350 mm^2^) compared to trabeculectomy with MMC (mitomycin-C) at 5 years follow-up^1,2^. The Ahmed valve implant is a GDD introduced in 1993 that has a valve mechanism^3^, which Coleman and associates have shown to be safe and efficacious in lowering intraocular pressure (IOP) for glaucoma treatment^4^. According to The Ahmed versus Barveldt Study^5^ the Barveldt 350-group had a higher success rate than the Ahmed-FP7 group at 1 year, but required a greater number of interventions. On the other hand, Ahmed verus Baerveldt study^6^ study showed similar success rates between Baerveldt and Ahmed devices, however Ahmed was associated with less failures due to safety issues at 5 years follow-up.

A main limitation of the Ahmed Glaucoma Valve (AGV; New World Medical, Inc., Rancho Cucamonga, CA) in comparison with other devices is a higher incidence of hypertensive phase. This occurs in 40–80%^7,8^ of patients and is thought to be due to bleb fibrosis and inflammation. It seems to be an unfavourable prognostic sign and may actually represent imminent failures^9^. Another tube-specific complication is the development of bleb encapsulation^10^. To try to avoid these complications antimetabolites, such as intraoperative MMC, has been used with controversial results^11–13^.

Ologen (Aeon Astron Corporation, Taipei, Taiwan) is a biodegradable collagen porous matrix that was developed aiming to replace MMC for trabeculectomy^14,15^. When placed under the conjunctiva, it not only acts as a reservoir but also helps to separate mechanically the conjunctiva from the valve’s plate and prevent adhesions between them^16^. This technique directs wound healing towards tissue regeneration and away from scar formation by guiding the patterns of fibroblasts migration^17^.

The aim of the study was to compare the clinical efficacy and safety of Ologen in AGV surgery. To the best of our knowledge this is the first randomized prospective multicentre clinical trial to evaluate Ologen with AGV implantation as compared with standard surgery, with the longest follow-up, and in Caucasian population.

## Materials and Methods

A multicenter, prospective, randomized clinical trial that enrolled 58 patients from 10 Spanish centers was designed. The study was approved by the institutional review board of the Hospital Clínico San Carlos of Madrid (Spain) and the design followed the tenets of the Declaration of Helsinki. A total of 58 glaucoma eyes, with indication of AGV (FP7 model) surgery were included, automatically randomized (www.randomization.com) and followed for 12 months. In the study group (27 patients) a 12 mm Ologen collagen matrix was associated to the AGV implantation and in the control group (31 patients) the conventional procedure was performed. The exclusion criteria were age less than 18 years or more than 85 years, allergic reaction to collagen, non light perception, other drainage implant in the same eye or scleral buckle or need of other surgery associated to the drainage implant.

After providing adequate explanations about the procedure a written informed consent was signed by all patients. Preoperatively, all subjects underwent a full ophthalmologic examination, including best-corrected visual acuity (BCVA), Goldmann (Haag-Streit AG, Gartenstadtstrasse 10, 3098 Koeniz, Switzerland) or Perkins applanation (Clement-Clarke, Haag-Streit, UK) tonometry (GAT), fundus examination, Humphrey visual field (Carl Zeiss Meditec, Dublin, CA, USA) and central corneal thickness (Dicon P55; Paradigm Medical Industries Inc., Salt Lake City, UT). Postoperatively GAT, BCVA and fundus examination were performed.

### Surgical Procedure

For both groups a fornix- based flap of the conjunctiva and Tenon’s capsule is performed in the superotemporal quadrant. The AGV body was then placed at least at 9 mm from the limbus and sutured to the sclera with a 9-0 nylon. A 23-gauge needle tract was used to enter the anterior, posterior or vitreous (after vitrectomy) chamber. The tube tip was cut obliquely, placed trough the tract and a donor sclera patch graft was secured with 10-0 nylon sutures over the exposed portion of the tube. In the study group a 12 × 1 mm Ologen was placed over the plate. Conjunctiva was sutured either with 8-0 or 9-0 Vicryl or with 10-0 Nylon sutures. The anterior chamber was reformed with BSS through a paracentesis tract and viscoelastic material was injected into the anterior chamber at the surgeon’s discretion.

### Postoperative Management

The postoperative follow-up visits were performed on days 1 and 7 and months 1, 3, 6 and 12. Eye drops consisting of Tobramycin and Dexametasone (Alcon Laboratories) were begun on the day after surgery and continued each 2 hours for the first week and descending doses attending to the degree of inflammation. If the examiner considered the intraocular pressure (IOP) was not controlled antiglaucoma eye drops were prescribed and added in this order: beta-blocker twice a day, alpha agonist brimonidine drops twice a day and carbonic anhydrase inhibitor twice a day. If IOP was not controlled a fixed combination of beta-blocker (brimonidine or carbonic anhydrase inhibitor) was prescribed.

### Outcome Measures

The primary surgical outcomes were postoperative IOP level and number of anti-glaucoma medications. Complete success was defined as an IOP equal or less than the target IOP without any anti-glaucoma medications. Two target IOP levels for complete success at 12 months were considered: IOP of ≤21 mmHg (target IOP 1) or ≤18 mmHg (target IOP 2). Qualified success was defined as an IOP of ≤21 mm Hg with/without anti-glaucoma medications. Failure was defined as IOP > 21 mmHg or <6 mmHg, IOP reduction at least of 20% from baseline, need of other procedure for IOP control or presence of devastating complications (endophthalmitis, lost of light perception, retinal detachment or suprachoroidal haemorrhage). A hypertensive phase was defined as an IOP > 21 mmHg within the first 3 months after surgery not being caused by tube obstruction or valve malfunction.

### Statistical analysis

Statistical analyses were performed using the SPSS software (version 15.0; SPSS, Inc., Chicago, IL, USA). For comparisons normally distributed numerical variables, unpaired/paired Student t tests and Mann-Whitney’s test was used for independent non-normally distributed variables. χ^2^ (or Fisher’s exact test) was used for qualitative variables. Kaplan-Meier survival analysis was performed to determine success rates and Log-rank test for the analysis. P values less than 0.05 were considered statistically significant.

Sample size was calculated to detect a difference between groups of 3 mmHg with an 80% power and an alpha error of 0.05, being necessary to include 25 patients en each group. To compensate post-randomization losses, at least 27 patients were included in each group.

## Results

58 Caucasian patients were included in the trial, 31 were randomized to the control group and 27 to the study group. There were 2 dropouts during the trial (one of each group, in the Ologen group after surgery and in the control group at day 1) due to causes beyond the study. Demographic features and basal data of the population are summarized in Table 1. No significant differences were observed between the groups.

**Table 1:** Patient demographic and basal characteristics.

	Control (n = 31)	Ologen (n = 27)	P
Mean age (years ± SD)	66,97 ± 18,72	57,78 ± 17,64	0,061**
Sex (% male)	41,93%	59,3%	0,188*
Eye (% right eyes)	51,6%	48,1%	0,792*
**Type of glaucoma**			
Primary	10 (32,3%)	10 (37%)	0,416*
Secondary	21 (67,7%)	16 (59,3%)	
**Types of secondary glaucoma**			
Pseudoexfoliative	1 (3,2%)	0	
Pigmentary	1 (3,2%)	1 (3,7%)	
ICE	1 (3,2%)	0	
Post-surgery	4 (12,9%)	2 (7,4%)	
Inflammatory	4 (12,9%)	4 (14,8%)	
Steroids	0	0	
Traumatic	2 (6,5%)	0	
Neovacular	5 (16,1%)	3 (11,1%)	
Not specified	3 (9,7%)	6 (22,2%)	
**Lens status**			
Phakic	8 (25,8%)	4 (14,8%)	0,336*
Aphakic/pseudophakic	23 (74,19%)	22 (81,5%)	0,792*
**Previous**			
Glaucoma surgery (trabeculectomy)	15 (48,4%)	14 (51,9%)	0,097*
Trabeculoplasty	3 (9,7%)	0	
Preoperative IOP(Mean ± SD)	30,96 ± 9,77	31,84 ± 8,32	*0*,718*
**No**. **Of preoperative medications**			
Mean (SD)	3 ± 0,82	2,73 ± 0,83	*0*,224**
Median (range)	3 (2–3)	3 (2–3)	
Oral acetazolamide	16 (51,6%)	20 (74,1%)	0,079*
Corneal thickness (μm)	539,36 ± 39,119	547,04 ± 34,291	0,459**
Visual field (MD)	−20,62 ± 11,33	−16,51 ± 8,1	0,265**

*χ^2^.

**T- Student.

MD: Mean Deviation.

**Figure 1:**
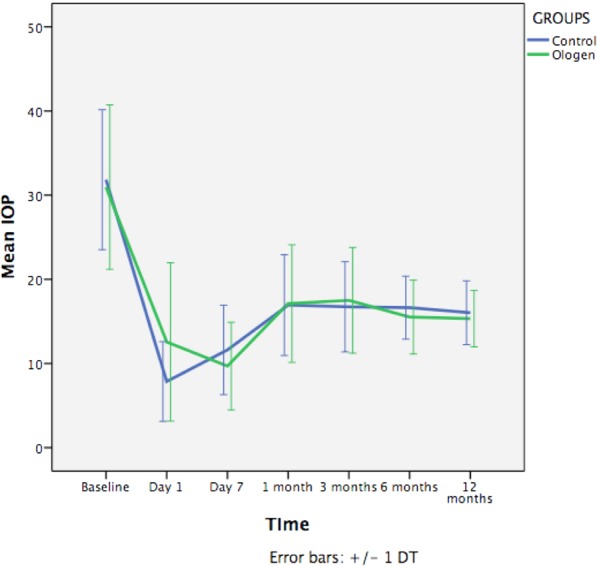
Represents the mean IOP during the follow-up period in the control and Ologen group.

**Figure 2:**
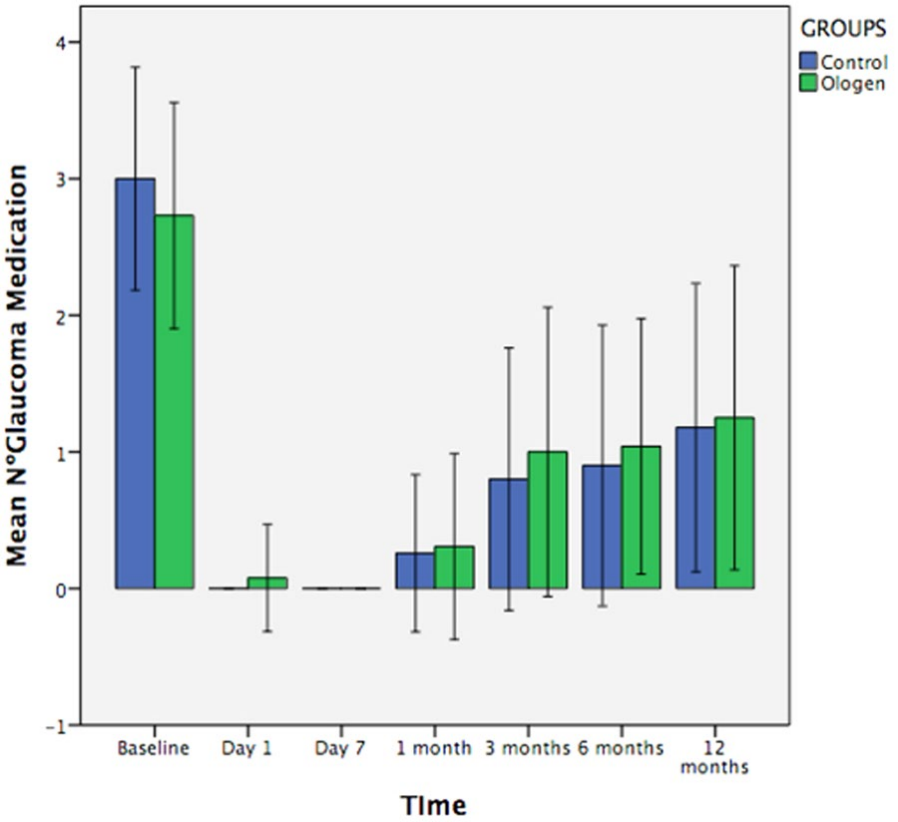
Shows number of glaucoma antiglaucoma medications in control and study group.

**Figure 3:**
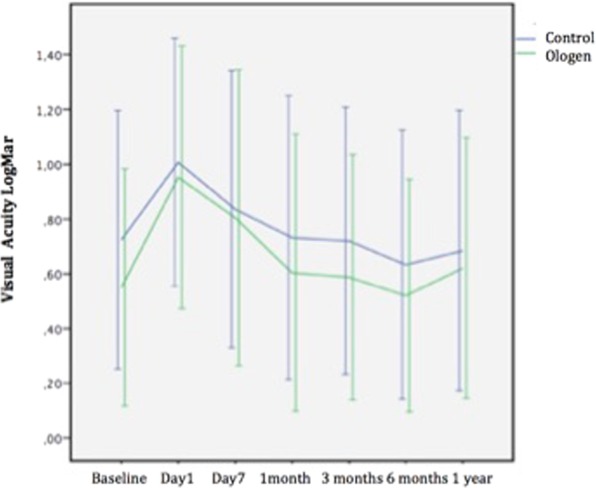
Visual acuity: baseline and follow-up in both groups.

**Figure 4:**
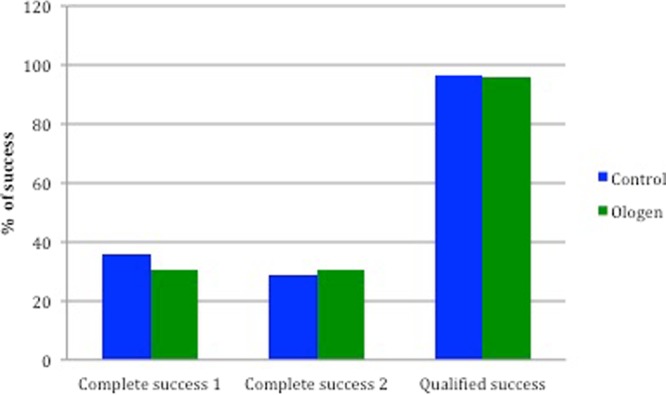
Percentages of complete and qualified success at 12 months.

**Figure 5:**
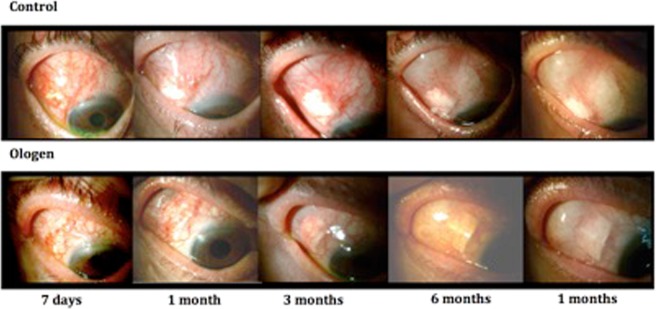
Photographs of surgical blebs in control and Ologen patients.

Figure 1 represents the IOP changes during the follow-up; the only significant difference between groups in the postoperative IOP was at day 1 (higher pressure in the Ologen group) (p = *0*,*022*). There was a significant difference of IOP between the baseline and all follow-up timings (p < 0,001). The number of glaucoma medications increased during the 12 months but never reached the number of previous treatments and no differences were found between groups (Fig. 2). Visual acuity in both groups during the follow-up is represented in Fig. 3, no differences were found between groups. There is a significant decrease in visual acuity in the first 24 hours and first week compared to baseline in both groups (Control 24 h p < 0,001 and day 7 p = 0,04; Ologen p = 0,0 24 hours and p = 0,003 day 7), and increases after the first week and no differences are found compared to baseline in the rest of the follow-up. 56% of patients in the control group and 55% in the Ologen group developed a hypertensive phase (p = 0,947). No differences were found in the complete or qualified success rates at 12 months (Fig. 4). Figure 5 shows photographs of two patient’s (control and Ologen patient) blebs during the follow-up.

**Figure 6:**
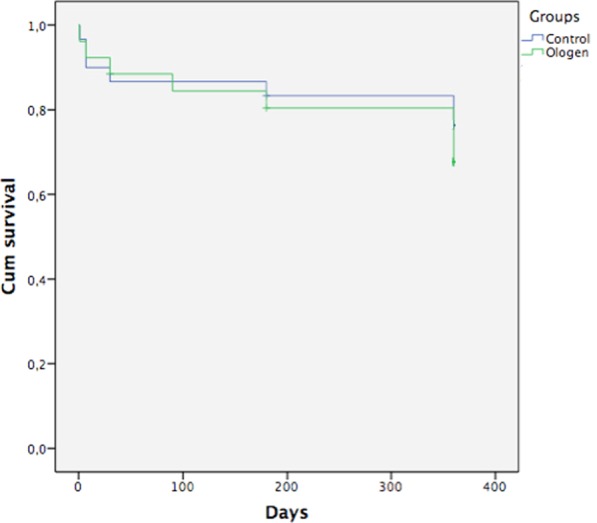
Kaplan-Meier success curve for both groups.

Kaplan-Meier curves showed no statistical difference between groups, success rates at 1 year were 76,7% in the control group and 69,2% in the Ologen group (Log Rank p = 0,531) (Fig. 6).

38,7% of patients in the control group suffered some complication during the follow-up and 61,5% in the Ologen group (p = 0,086), Table 2 shows details regarding the complications.

**Table 2:** Early and late complications in Control and Ologen group.

	Control Num. eyes (%)	Ologen Num. eyes (%)
**Complications**		
Early (<3 months)	31	26
Hyphema	3 (9,7%)	6 (23%)
Vitreous haemorrhage	1 (3,2%)	0
Shallow anterior chamber	2 (6,5%)	2 (7,7%)
Wound leak	1 (3,2%)	1 (3,8%)
Acute glaucoma	0	1 (3,8%)
Conjunctiva dehiscence	1 (3,2%)	1 (3,8%)
Choroidal detachment	0	2 (7,7%)
Posterior synechiae	1 (3,2%)	1 (3,8%)
Corneal dellen	1 (3,2%)	0
Strabismus	1 (3,2%)	0
Tube block	0	1 (3,8%)
Bleb encapsulation	0	2 (7,7%)
**Late (>3 months)**		
Cataract	3 (10%)	2 (8%)
Tube block	0	1 (4%)
Tube exposure	0	1 (4%)
Corneal dellen	1 (3,3%)	0
Decreased vision (>1 line)	4 (13,3%)	3 (12%)

## Discussion

Our study showed that the use of Ologen in AGV surgery did not offer better IOP control, less need of antiglaucoma medications or higher success rates than conventional surgery after one-year follow-up.

Valved GDDs provide a good early postoperative IOP control^4^ however; this control is frequently followed by a rise in IOP due to fibrosis and inflammation around the device that may result in surgical failure^9,10^. Minimizing or modulating the scarring process and fibrosis around the AGV plate should improve the surgical success. Ologen is a potential alternative method for controlling the wound healing process and for avoiding the complications of antifibrotic agents. Rho *et al*.^18^ evaluated a collagen matrix in AGV surgery and found promising results at 6 months in Asian population.

Our study only revealed IOP differences in the first postoperative day, showing lower values in the control group. This difference may be due to the resistance effect of the collagen matrix to the aqueous outflow during the first hours as described by DeCroos^19^, however Rho *et al*.^18^ did not find such difference that may be probably due to the tube ligature.

This group found better IOP control at one month in the study group and less number of postoperative antiglaucoma medications. However, we did not find differences in the number of medications during the follow-up. Our results in IOP control and number of antiglaucoma medications in both groups were comparable to other results reported using AGV^4,10,20–22^.

Visual acuity after surgery is decreased the first weeks in both groups normally due to inflammation and hyphema but is not affected at 12 months after surgery.

A hypertensive “phase” was depicted in 56% in the control group and 55% in the study group, although these are fairly high percentages both are in the range of what is found in AGV reports that lie between 30–80%^10,23,24^. These results also differ from those registered by Rho *et al*.^18^, they found an incidence of hypertensive phase in 48% of patients in the control group and 4,5% with collagen matrix implantation^18^. They discuss that there is a similar time course between the hypertensive phase and Ologen’s *in vivo* maintenance period(3–6months), they speculate that during this time fibroblasts enter the porous collagen structure before it degrades and this would offer a larger bleb volume preventing the hypertensive phase. Our results, however, cannot support this hypothesis. The main differences between both are the population included and the surgical technique. Rho *et al*.^18^ included patients without prior glaucoma surgeries who may have more favourable healing process, and they ligated the tube which delays early contact of AH with the conjunctiva and could reduce conjunctival fibrosis^10,25,26^.

We found no differences between groups in the complete or qualified success, or in the survival rate at 12 months. Again, Rho *et al*.^18^ found significant better success results in the collagen matrix group, but with a shorter follow up that make results not so comparable.

It is difficult to compare survival rate results between studies because of the different failure/success criteria and different follow-up time. In both our groups, the main reason for failure was the inadequate IOP control as it occurs in other AGV studies^22,24^.

Although the number of complications was higher in the Ologen group, the difference was not statistically different. No complication was so serious to threaten vision; the most common early complications were hyphema and shallow anterior chamber as registered in other studies^4,23,24^. One eye in the Ologen group developed a large Tenon’s cyst, which progressively enlarged and was surgically excised.

The collagen matrix Ologen is supposed to help the scarring process and reduce the fibrous encapsulation around the AGV plate, which would reduce medium and long-term IOP elevation and decrease the rate of hypertensive phase. But in contrast to the results observed by Rho *et al*.^18^ we did not find advantages in the use of this collagen matrix in AGV surgery.

In this controlled clinical trial patients had similar demographics and baseline characteristics. However, one limitation is the limited follow-up as 1 year might be not enough to reveal possible differences in the long-term success and long-term complications. Also, the limited number of patients may not be enough to prove differences between groups.

In summary, this clinical trial shows that although Ologen collagen matrix in AGV surgery seems to be safe with no significant added risk, and it is not associated with higher success rate or a lower incidence of hypertensive phase.
